# Left upper lobectomy can be a risk factor for thrombosis in the pulmonary vein stump

**DOI:** 10.1186/1749-8090-9-5

**Published:** 2014-01-06

**Authors:** Kazuto Ohtaka, Yasuhiro Hida, Kichizo Kaga, Yasuhiro Takahashi, Hiroshi Kawase, Satoshi Hayama, Tatsunosuke Ichimura, Naoto Senmaru, Naotake Honma, Yoshiro Matsui

**Affiliations:** 1 Department of Thoracic Surgery, Steel Memorial Muroran Hospital, 1-45 Chiribetsu-cho, Muroran, Hokkaido 050-0076, Japan; 2 Department of Cardiovascular and Thoracic Surgery, Hokkaido University Graduate School of Medicine, Sapporo, Hokkaido Japan

**Keywords:** Left upper lobectomy, Pulmonary vein stump, Thrombosis, Cerebral infarction

## Abstract

**Background:**

Thrombosis in the left upper pulmonary vein stump after left upper lobectomy is a very rare but important complication because it occurs in the systemic circulation system. We previously made the first ever report on the frequency and risk factors of thrombosis in the pulmonary vein stump after lobectomy. In this study, we conducted an investigation in a different hospital to determine whether this was a common complication.

**Methods:**

From 2008 to 2012, 151 patients who underwent lobectomy and following enhanced CT within 2 years after the operation were studied. Postoperative contrast-enhanced CT imaging was retrospectively checked.

**Results:**

We found thrombosis in the pulmonary vein stump in 5 of the 151 patients (3.3%). All 5 patients underwent left upper lobectomy (17.9% of the patients who underwent left upper lobectomy). These 5 patients did not have infarction of any vital organ. The thrombus was disappeared several months later on contrast-enhanced CT in 3 patients and followed in 2 patients. On univariate analysis, there was a significant difference only in the operative procedure (p < 0.001).

**Conclusions:**

Thrombosis in the pulmonary vein stump occurred with high frequency in patients who underwent left upper lobectomy. Because the frequency of thrombosis in this study was the same as in our previous report, this might be a common complication.

## Background

We previously experienced a case of cerebral infarction with thrombosis in the pulmonary vein (PV) stump that was demonstrated on enhanced CT after left upper lobectomy (LUL). Thereafter, we experienced two other cases with thrombosis in the PV stump after lobectomy without cerebral infarction, and reported these 3 cases [1]. There have been reported 3 cases of thrombosis in the PV stump on chest CT after lobectomy that developed infarction of vital organs as well as 3 other cases with infarction of vital organs after lobectomy but no thrombosis on chest CT [2-6]. All 6 of those patients and our 3 patients underwent LUL. Therefore, we hypothesized that LUL was a risk factor for thrombosis in the PV stump, which could cause infarction of vital organs after lobectomy.

We previously reported a retrospective study that checked the PV stump on postoperative enhanced CT in 193 non-small cell lung cancer (NSCLC) patients who underwent lobectomy [7]. In that study, thrombosis in the PV stump after lobectomy was detected in 7 patients (3.6%), all of whom underwent LUL. Among the patients who underwent LUL, thrombosis was detected in 13.5%. One of the 7 patients developed cerebral infarction. Our previous report was the first study on thrombosis in the PV stump after lobectomy and clarified that this occurred with a high frequency after LUL.

To determine whether this was a common complication, we conducted the present study on thrombosis in the PV stump after lobectomy in another hospital.

## Methods

The Muroran Steel Memorial Hospital Institutional Review Board approved this retrospective study and waived the requirement for informed concent.

### Patients

Of the 203 consecutive patients who underwent lobectomy from April 2008 to December 2012 in Muroran Steel Memorial Hospital, 151 patients who underwent contrast-enhanced chest-CT at least once within 2 years after the lobectomy were selected. Contrast-enhanced chest CT images for postoperative follow-up in these patients were retrospectively interpreted by a general thoracic surgeon to check for thrombosis in the PV stump. The clinicopathological data were obtained from the medical charts. The pathological staging was based on the 7th edition of the Union Internationale Contra le Cancer (UICC) staging system on tumor-node-metastasis (TNM) classification.

### Operative procedure

Video-assisted thoracoscopic surgery (VATS) or hybrid VATS was performed for all cases except those with large tumor size, chest wall invasion, bronchial invasion, or pulmonary vessel invasion.

Lobectomy was performed as follows. First, the PV was divided using a linear stapler. If the PV could not be divided at that time, one or two branches of it were divided with ligation and the rest of the PV was divided using a linear stapler. Second, a few branches of the pulmonary artery (PA) were divided either by ligation or with a linear stapler. Finally, the bronchus was divided using a linear stapler.

### Postoperative adjuvant chemotherapy

Adjuvant chemotherapy was indicated for all patients who gave informed consent except for the patients with Stage IA. Some of the patients with pT1bN0M0 stage IA took UFT orally for 2 years postoperatively. Other patients underwent intravenous chemotherapy. The patients who discontinued UFT within 2 months and those who discontinued intravenous chemotherapy within 1 cycle were classified into the group without chemotherapy for analysis.

### Statistical analysis

Univariate analyses were performed using the Mann–Whitney U test or Fisher’s exact test. All statistic analyses were performed using Stat Flex ver. 6.0 software (Artech Co., Ltd., Japan). A p value of less than 0.05 was regarded as statistically significant.

## Results

The 151 patients consisted of 101 males and 50 females with a median age of 72 years (range: 38 to 82 years old). Past histories consisted of 79 cases of hypertension, 39 malignant diseases, 22 cases of diabetes mellitus, 19 ischemic heart diseases, 15 arrhythmias, and 15 cerebral vascular diseases. Antithrombotic drugs were taken by 38 patients.

The operative procedures consisted of 54 right upper lobectomies (RUL), 9 right middle lobectomies (RML), 30 right lower lobectomies (RLL), 28 LUL, and 30 left lower lobectomies (LLL). The operative approaches were 103 VATS, 14 hybrid VATS, and 34 open thoracotomies. The median operative time was 296 minutes (range: 143 to 482 minutes). The median amount of blood loss was 65 ml (range: 0 to 1500 ml). The median postoperative hospital stay was 10 days (range: 5 to 179 days). Postoperative complications developed in 45 patients.

There were 139 cases of primary lung cancer, 10 of metastatic lung tumors, and 2 benign tumors. Of the primary lung cancers, there were 87 cases of adenocarcinoma, 35 of squamous cell carcinoma, 6 of adenosquamous cell carcinoma, 5 of small cell carcinoma, and 6 others. The median tumor size was 25 mm (range: 9 to 67 mm). Postoperative adjuvant chemotherapy was performed for 44 patients.

### Thrombosis in the PV stump after lobectomy

**Figure 1:**
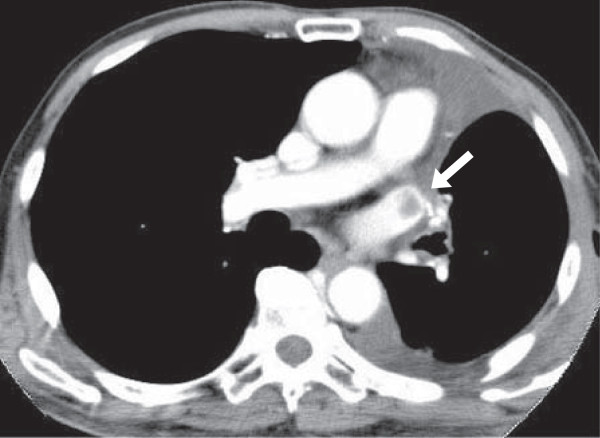
**A thrombus in the left superior pulmonary vein stump after left upper lobectomy.** Contrast-enhanced CT on a 78 year-old male to check for other diseases on postoperative day 22 shows a thrombus in the LSPV stump.

**Figure 2:**
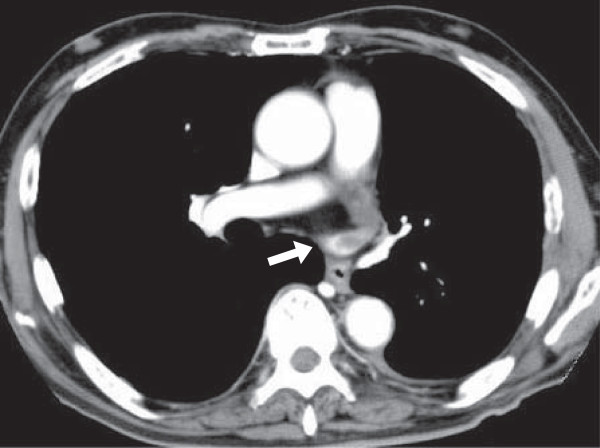
**A thrombus in the left superior pulmonary vein stump after left upper lobectomy.** Contrast-enhanced CT on a 66 year-old male for follow-up 7 months postoperatively shows a thrombus in the LSPV stump. This thrombus was not detected at that time.

Thrombosis in the PV stump after lobectomy developed in 5 of the 151 patients (3.3%), all of whom had undergone LUL, and who accounted for 5 of the 28 patients who underwent that procedure (17.9%). The thrombosis were found on contrast-enhanced CT for check-up of other disease within a month postoperatively in 2 patients and on contrast-enhanced CT for follow-up at 6 to 7 months postoperatively in 3 patients (Figures 1 and 2). These 5 patients did not have arrhythmia as atrial fibrillation and then infarction of any vital organ. In 2 patients, anticoagulant therapy was immediately started and CT a few months after the operation showed that the thrombus had disappeared. Then, anticoagulant therapy was stopped. In a patient, the thrombosis was not found at that time and disappeared after 9 months without any treatment. In 2 patients, the thrombosis is followed now with anticoagulant therapy.

### Risk factors for thrombosis in the PV stump after lobectomy

Clinicopathological factors were compared between the 5 patients with thrombosis in the PV stump and the 146 patients without thrombosis (Table 1). Only the operative procedure showed a significant difference (p < 0.001). Comorbidities, operative factors, and pathological factors did not show a significant difference.

**Table 1:** Univariate analyses of clinicopathological factors associated with thrombosis in the pulmonary vein stump

	**Patients with thrombosis (n = 5)**	**Patients without thrombosis (n = 146)**	**P value**
Age, median value (range), years old	75 (56–81)	72 (38–82)	0.5052
Sex, n (%)			
Male / Female	4 (80.0) / 1 (20.0)	97 (66.4) / 49 (33.6)	0.4631
Brinkman index, median value (range)	750 (0–1200)	600 (0–3180)	0.6092
Comorbidity, n (%)			
History of malignant disease	2 (40.0)	37 (25.3)	0.3849
Hypertension	2 (40.0)	77 (52.7)	0.4560
Diabetes mellitus	0 (0)	22 (15.1)	0.4498
Cerebral infarction	0 (0)	11 (7.5)	0.6815
Arrhythmia	0 (0)	15 (10.3)	0.5883
Cardiovascular disease	0 (0)	19 (13.0)	0.5055
Anti-coagulate drug	0 (0)	38 (26.0)	0.2294
Steroids	0 (0)	3 (2.1)	0.9033
Operative approach, n(%)			
Thoracotomy / VATS	4 (80.0) / 1 (20.0)	113 (77.4) / 33 (22.6)	0.6858
Procedure, n (%)			
RUL, RML	0 (0)	63 (43.2)	<0.001
RLL	0 (0)	30 (20.5)	
LUL	5 (100)	23 (15.8)	
LLL	0 (0)	30 (20.5)	
Operative time, median value (range), minutes	301 (240–425)	295 (143–482)	0.8069
Dissection of mediastinal lymph nodes, n (%)	4 (80.0)	132 (90.4)	0.4117
Blood loss, median value (range), ml	90 (50–750)	60 (0–1500)	0.2082
Duration of postoperative drainage, median value (range), days	4 (2–4)	4 (2–12)	0.6149
Postoperative hospital stay, median value (range), days	15 (7–28)	10 (5–179)	0.1828
Postoperative complication, n (%)	2 (40.0)	38 (26.0)	0.3990
Diagnosis, n (%)			
Primary / Metastasis / Benign	5 (100) / 0 (0) / 0 (0)	134 (91.8) / 10 (6.8) / 2 (1.4)	0.7999
Pathological stage, n (%)			
I / II / III	4 (80) / 0 (0) / 1 (20)	97 (66.4) / 14 (9.6) / 21 (14.4)	0.7378
Adjuvant chemotherapy, n (%)	1 (20.0)	44 (30.1)	0.5310

## Discussion

In this study, thrombosis in the PV stump developed in 3.3% of the patients who underwent lobectomy, and in 17.9% of those who underwent LUL. We previously reported that thrombosis developed in 3.6% of patients who underwent lobectomy and in 13.5% of those who underwent LUL [7]. Because the frequencies of thrombosis in the PV stump after lobectomy and after LUL were nearly equal in the two studies, this might be a common complication after LUL. Though this complication is not well known, it is very important because thrombosis in the systemic circulation might cause infarction of vital organs.

Clinically, thrombi in the systemic circulation typically develop as a result of atrial fibrillation. It is reported that left lobectomy might be a risk factor for atrial fibrillation [8]. However, of the 5 patients with thrombosis in this study, the 7 patients with thrombosis in our past study, and 6 patients in the past case report, none had atrial fibrillation when the thrombosis was diagnosed [2-7]. Moreover, all had undergone LUL, and none LLL. Therefore, the probability that atrial fibrillation or left lobectomy caused the thrombosis in the PV stump after lobectomy was low, whereas there was a high probability that LUL caused it.

In a previous study, we reported risk factors for thrombosis in the PV stump after lobectomy [7]. On univariate analysis comparing groups of patients with and without thrombosis in the PV stump, LUL and operative time showed significant differences, while postoperative adjuvant chemotherapy showed a marginal difference. In this study, there was a significant difference only for the operative procedure (Table 1). Based on our 2 studies in 2 institutes, LUL might be a risk factor for thrombosis in the PV stump after lobectomy.

**Figure 3:**
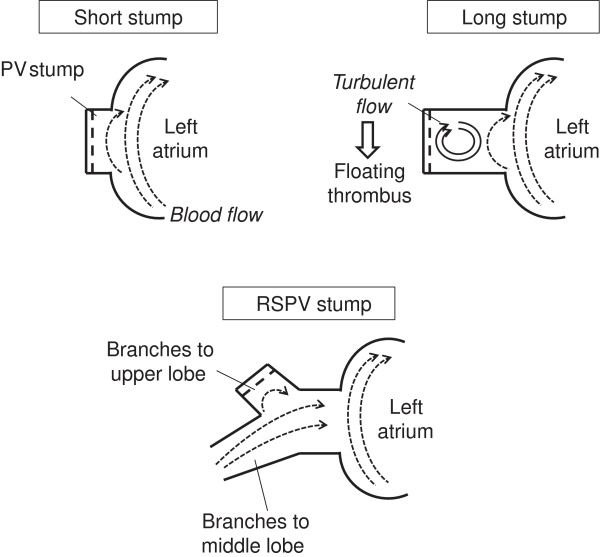
**The hypothesis of a thrombus in the pulmonary vein stump after lobectomy.** In a short PV stump, blood flow may occur because that in the left atrium spreads throughout the stump. In the long PV stump, turbulent flow or stasis of blood may occur because blood flow in the left atrium does not spread throughout the stump.

We speculated that the cause of thrombosis in the left superior PV (LSPV) stump after LUL was the long LSPV stump. It might develop because turbulent flow or stasis of blood occurs in the long PV stump. Kwek et al. reported that thrombosis developed in longer PA stumps [9]. In the short PV stump, blood flow may occur because that in the left atrium spreads through the entire PV stump. In the long PV stump, turbulent flow or stasis of blood may occur because blood flow in the left atrium does not spread throughout the PV stump (Figure 3). In the right superior PV, because the branches to the upper lobe and middle lobe remain after RUL or RML, blood flow in the remaining branches spreads throughout the stump and turbulent flow or stasis of blood may not occur. In a previous report, we compared the lengths of 4 PV stumps after lobectomy using three-dimensional CT images [7]. As a result, it was demonstrated that the LSPV stump remained significantly longer than the other 3 PV stumps. Intrapericardial LSPV may be anatomically longer than the other 3 PVs. However, it has not yet been clarified whether turbulent flow or stasis of blood occurs and causes thrombosis in the long LSPV stump.

Why have there so few reports on thrombosis in the PV stump after lobectomy? We speculate that the reason may be that many institutes perform postoperative follow-up using chest X-rays or plane CT. Most guidelines recommend follow-up with plane CT or without CT after lung surgery [10]. If patients who undergo lobectomy are followed using contrast-enhanced CT and doctors conducting follow-up observe the PV stump on contrast-enhanced CT images, more patients with thrombosis in the PV stump may be detected.

At this time, we would propose the following measures with regard to thrombosis in the PV stump after lobectomy. First, the LSPV stump should be as short as possible. For example, after the LSPV is divided using a linear stapler, a ligation can be added to the LSPV stump near the pericardium to shorten it. Second, for early detection of possible thrombosis in the LSPV, contrast-enhanced CT must be performed as early as possible at least one time after LUL. Because it is difficult to demonstrate the PV stump by echocardiography after lobectomy, we recommend contrast-enhanced CT. Transesophageal echocardiography is useful, too. However transesophageal echocardiography may be painful test and more invasive than enhanced-CT because transesophageal echocardiography sometimes has a need for sedation. Third, if a thrombus in the LSPV stump is detected without an embolism, anticoagulant therapy should immediately be started. It seems to be premature to give thrombolytics because the benefit from it may not be greater than the risk of complications from it.

There are many questions regarding thrombosis of the PV stump. How often does this thrombosis generally occur? How often does this thrombosis cause infarction of vital organs? Is the risk of infarction of vital organs caused by this thrombosis higher than the risk of hemorrhagic event caused by anticoagulant therapy? Because there have been few reports about thrombosis in the LSPV stump after LUL, there is a pressing need for study in many institutes to clarify the frequency and risk factors for this complication. It is also an important issue that how aggressively the PV thrombi should be treated to prevent arterial infarctions.

## Conclusions

In this study, it was clarified that the thrombosis in the LSPV stump after LUL was a common complication that potentially results in arterial thromboembolism.

## Abbreviations

PV: Pulmonary vein; LUL: Left upper lobectomy; NSCLC: Non-small cell lung cancer; VATS: Video-assisted thoracoscopic surgery; PA: Pulmonary vein; RUL: Right upper lobectomy; RML: Right middle lobectomy; RLL: Right lower lobectomy; LLL: Left lower lobectomy; LSPV: Left superior pulmonary vein.

## Competing interests

The authors declare that they have no competing interests.

## Authors’ contributions

KO performed the study design, the acquisition of data, and the statistical analysis, and drafted the manuscript. YH, KK, YT, HK, SH, TI, NS, NH, and YM helped to draft the manuscript. All authors read and approved the final manuscript.
